# Fast and interpretable genomic data analysis using multiple approximate kernel learning

**DOI:** 10.1093/bioinformatics/btac241

**Published:** 2022-06-27

**Authors:** Ayyüce Begüm Bektaş, Çiğdem Ak, Mehmet Gönen

**Affiliations:** Graduate School of Sciences and Engineering, Koç University, İstanbul 34450, Turkey; Knight Cancer Institute, Oregon Health & Science University, Portland, OR 97239, USA; Department of Industrial Engineering, College of Engineering, Koç University, İstanbul 34450, Turkey; School of Medicine, Koç University, İstanbul 34450, Turkey

## Abstract

**Motivation:**

Dataset sizes in computational biology have been increased drastically with the help of improved data collection tools and increasing size of patient cohorts. Previous kernel-based machine learning algorithms proposed for increased interpretability started to fail with large sample sizes, owing to their lack of scalability. To overcome this problem, we proposed a fast and efficient multiple kernel learning (MKL) algorithm to be particularly used with large-scale data that integrates kernel approximation and group Lasso formulations into a conjoint model. Our method extracts significant and meaningful information from the genomic data while conjointly learning a model for out-of-sample prediction. It is scalable with increasing sample size by approximating instead of calculating distinct kernel matrices.

**Results:**

To test our computational framework, namely, Multiple Approximate Kernel Learning (MAKL), we demonstrated our experiments on three cancer datasets and showed that MAKL is capable to outperform the baseline algorithm while using only a small fraction of the input features. We also reported selection frequencies of approximated kernel matrices associated with feature subsets (i.e. gene sets/pathways), which helps to see their relevance for the given classification task. Our fast and interpretable MKL algorithm producing sparse solutions is promising for computational biology applications considering its scalability and highly correlated structure of genomic datasets, and it can be used to discover new biomarkers and new therapeutic guidelines.

**Availability and implementation:**

MAKL is available at https://github.com/begumbektas/makl together with the scripts that replicate the reported experiments. MAKL is also available as an R package at https://cran.r-project.org/web/packages/MAKL.

**Supplementary information:**

Supplementary data are available at *Bioinformatics* online.

## 1 Introduction

Cancer is one of the most challenging healthcare problems of our era. With increasing size of patient cohorts and genomic characterizations collected from tumour biopsies, machine learning algorithms have been applied to different tasks in the hope of discovering molecular mechanisms related to the diagnosis, prognosis and treatment of cancer. Together with good predictive performance, building interpretable machine learning algorithms plays a crucial role in cancer research to discover new therapeutic biomarkers and to analyze the heterogeneity of tumours. Kernel-based machine learning algorithms have been widely used in computational biology, thanks to their capability to deal with highly correlated data and interpretability. However, the problem with these existing algorithms is their lack of scalability, which started to cause problems especially for single-cell cancer research, since traditional kernel-based methods are computationally prohibitive with large sample sizes.

Kernel machines attract a significant amount of attention since they are able to approximate any function during the learning process when the size of the training data is large enough. Kernel machines are also capable of incorporating prior knowledge into learning process using the kernel function. However, machine learning methods that need to calculate a kernel matrix over the dataset scale poorly with increasing training sample size, since the size of the kernel matrix is quadratic in the number of training samples. One of the most widely known kernel-based machine learning method is the support vector machine algorithm (Boser *et al.*, 1992; Cortes and Vapnik, 1995; Guyon *et al.*, 1993).

The integration of kernel matrices into a linear model to solve a non-linear problem called the ‘kernel trick’. A kernel can be described as a measure of similarity between data points, which corresponds to a dot product in some implicit feature space (Schölkopf and Smola, 2002). The kernel trick basically enables us to map the input data to a higher dimensional feature space and then to solve the originally nonlinear problem in this high-dimensional and implicit feature space.

Instead of learning with a single kernel matrix, one may choose to learn with multiple kernel matrices calculated using different kernel functions on the same data representation, using the same kernel function on different data partitions or using same/different kernel functions on different data representations, which is known as multiple kernel learning (MKL). Some of the existing MKL algorithms assign equal weights to the input kernels, which may not be the best way of discovering the underlying dynamics. For instance, in computational biology, to discover the underlying molecular mechanisms from genomic data, instead of initially setting the kernel weights, making the MKL algorithm choose the weights assigned to each kernel in a data-dependent manner is more convenient. Additionally, combining kernels in a non-linear or data-dependent way is stated to seem more promising than linear combination in fusing information provided by simple linear kernels, whereas linear methods are stated as being more reasonable in case of combining complex Gaussian kernels (Gönen and Alpayd, 2011).

The main contribution of this study is to extend well-known MKL idea to large-scale problems using an approximation procedure for kernel matrices. The proposed algorithm was shown to have four important characteristics: (i) running in a scalable manner on large-scale genomic data, (ii) getting sparse solutions, (iii) integrating prior information during the learning process to increase the interpretability and (iv) maintaining or even increasing the predictive performance.

## 2 Materials

In this study, we used three datasets formed using two data sources. First, we used data from The Cancer Genome Atlas (TCGA) project, to discover the molecular mechanisms related to early- and late-stage cancers and 2-year survival of patients. Second, we used single-cell RNA-Seq data provided by the Broad Institute Single Cell Portal to understand molecular signatures related to cell malignancy, which could be beneficial for understanding tumour plasticity and cancer progression mechanisms.

### 2.1 TCGA dataset

We gathered genomic data and clinical annotation files of over 10 000 cancer patients from 33 cancer cohorts in the Genomics Data Commons (GDC) data portal offered by TCGA consortium at https://portal.gdc.cancer.gov. TCGA shared the RNA-Seq measurements of the tumours from 33 cohorts and pre-processed them with a unified pipeline. We downloaded HTSeq-FPKM profiles of all primary tumours from the most recent data freeze (i.e. Data Release 31—October 29, 2021) and finally got 9911 files in total. To obtain tumour grade levels and survival-related information, we used clinical annotation files of the patients.

For binary classification task of cancer stages, we considered patients clinically annotated as Stage I as having early-stage cancer; patients clinically annotated with Stages II, III or IV as having late-stage cancer. We included cohorts having at least 20 tumours from both categories. We combined data coming from all cohorts to form one big dataset of 19 814 features. As a result, the final dataset contains 5547 patients coming from 15 distinct cohorts.

For 2-year survival classification, we considered patients that have vital_status, days_to_death, days_to_last_followup, days_to_last_known_alive information. We combined data from 33 cohorts by including patients with the required survival information. We included cohorts having at least 20 patients whose vital_status is dead and at least 100 patients in total. The resulting dataset contains 5168 patients coming from 20 distinct cohorts with 19 814 expression features. We labelled the patient as having ≥2-year survival (i) if vital_status is alive and days_to_last_followup or days_to_last_known_alive is ≥2 years; (ii) if vital_status is dead and days_to_death is ≥2 years. On the other hand, we labelled the patient as having <2-year survival if vital_status is dead and days_to_death is <2 years.

### 2.2 Single-cell RNA-Seq dataset

Analysis of single-cell RNA-Seq data holds promise to find new ways to treat cancer. To reveal the mechanisms related to tumour plasticity for targeted cancer therapies and to find new biomarkers, single-cell analysis is in the spotlight. Having this motivation, we searched for the datasets in the Broad Institute Single Cell Portal at https://singlecell.broadinstitute.org and found the melanoma immunotherapy dataset, which is appropriate for our binary classification task, provided by Jerby-Arnon *et al.* (2018) as a part of their research.

We intersected the gene expression profiles of 7186 cells with malignancy information coming from 6738 clinical annotation files. For each cell, there were 23 686 gene expression measurements in the dataset. If a cell is annotated as B.cell, CAF, Endothelial.cell, Macrophage, NK, T.CD4, T.CD8 or T.cell, we labelled this cell as non-malignant. All other cells were labelled as malignant.

### 2.3 Pathway/gene set collections

We used two pathway/gene set collections from the Molecular Signatures Database (MSigDB) that are specifically curated for cancer research: Hallmark gene set collection (Liberzon *et al.*, 2015) and Pathway Interaction Database (PID) pathway collection (Schaefer *et al.*, 2009), as prior information sources to be used for calculation of multiple approximate kernel matrices. The Hallmark gene set collection contains 50 gene sets (i.e. feature sets) with sizes varying between 32 and 200, whereas the PID collection include 196 pathways (i.e. feature sets) with sizes varying between 10 and 137.

## 3 Methods

To bypass the computationally expensive kernel matrix calculation step of MKL, we used approximation matrices instead of exact kernel matrices. After accelerating the kernel matrix computation step, we combined approximation matrices calculated for each pathway/gene set. We then fed the resulting combined matrix into group Lasso formulation, which led to sparsity in pathway/gene set level and gave interpretable results.

### 3.1 Multiple approximate kernel learning: fast multiple approximate kernel learning

In kernel-based methods, the idea of integrating kernel matrices into a linear model to solve a nonlinear problem is achieved by applying the ‘kernel trick’. The kernel trick is a result from the fact that any positive definite function $k(x_{i},x_{j})$ with $x_{i},x_{j}\in R^{d}$ defines an inner product and a mapping function Φ(·) so that the inner product between mapped data points can be quickly computed as $\langle \Phi (x_{i}),\Phi (x_{j})\rangle =k(x_{i},x_{j})$ (Schölkopf and Smola, 2002).

To accelerate the kernel matrix computation step of MKL, we used a modified version of random Fourier features mentioned by Rahimi and Recht (2008). Instead of using the implicit mapping by the aforementioned kernel trick, they proposed explicitly mapping the data to a low-dimensional Euclidean inner product space using a randomized feature map $z:R^{d}\to R^{D}$, so that the inner product between a pair of mapped points $z(x_{i})$ and $z(x_{j})$ approximates their kernel evaluation: $k(x_{i},x_{j})=\langle \Phi (x_{i}),\Phi (x_{j})\rangle \approx z{\left(x_{i}\right)}^{⊤}z(x_{j})$. This calculation of random features is notable since this new feature map z(·) is low-dimensional. In fact, the dimensionality of randomized feature space *D* is given as an input to algorithm, and it can cope with large-scale data well, since instead of calculating kernel matrices which are of size *N *×* N*, we now need to calculate matrices of size *N *×* D*, where D≪N.

Once the appropriate low-dimensional approximation method for the kernel matrices is set, another problem arises at how to integrate this approximation matrices into the learning process.

As a popular model selection and shrinkage estimation method, the Lasso estimator had been proposed by Tibshirani (1996). Then, the group Lasso has been proposed as an extension to the Lasso algorithm (Antoniadis and Fan, 2001; Bakin, 1999; Cai, 2001; Yuan and Lin, 2006). The group Lasso is the least-square regression problem with regularization by a block $\ell_{1}$-norm. This estimator works well in case of dealing with grouped data which in fact leads to the idea of MKL algorithm. Bach (2008) discussed theoretical aspects of the consistency of the group Lasso and using multiple kernel matrices.

Despite giving interpretable results, the problem with all ordinary MKL methods is their scalability issue owing to their need to calculate distinct kernel matrices, which scale quadratically with the sample size. This issue could be problematic once they are applied to large-scale datasets in areas such as computational biology, computer vision etc.

#### 3.1.1 Random features for kernel approximation

Owing to high computational complexity of using kernel trick in large-scale datasets, the questions of finding faster methods arises. Instead of computing the kernel matrices, to accelerate the learning process, there is a need to find a low-dimensional approximation for kernel matrix calculation. For this purpose, we approximate the kernel matrices using a modified version of random feature mapping originally has been introduced by Rahimi and Recht (2008). This approximation method is a natural result from a classical harmonic analysis as presented below.

Theorem 1. *A continuous kernel* $k(x_{i},x_{j})=\kappa (x_{i}-x_{j})$  *on* $R^{d}$  *is positive definite if and only if* κ(θ)  *is the Fourier transform of a non-negative measure (Rudin, 1994).*

Briefly, as a result of Bochner’s theorem, we are ensured that for any properly scaled shift-invariant kernel k(·,·), its Fourier transform is a proper probability distribution and $z{\left(x_{i}\right)}^{⊤}z(x_{j})$ is an unbiased estimate of $k(x_{i},x_{j})$ in case the random samples are drawn from the Fourier transform p(·). The Fourier transform p(·) for a given kernel κ(·) is:

(1) $$p(\omega )=\frac{1}{2\pi }\int e^{-j\omega^{⊤}\theta }\kappa (\theta )d\theta .$$

To calculate similarity between pairs of gene expression profiles, we used the Gaussian kernel on feature sets in Algorithm 1, which is a modified version of the random feature mapping algorithm proposed by Rahimi and Recht (2008). We approximated a given Gaussian kernel matrix by getting a low-dimensional approximation matrix **Z** as the output from this algorithm, where we used the sin(·) function in addition to the cos(·) function to guarantee that $ZZ^{⊤}$ produces a kernel matrix with ones on the diagonal. Due to this modification, we also changed the normalizing constant from $\sqrt{2/D}$ to $\sqrt{1/D}$.

The Gaussian kernel was previously reported in the literature as being reliable to be used with high-dimensional genomic data (Costello *et al.*, 2014; Gönen *et al.*, 2017). We used the same approach to discover highly nonlinear dependency between the RNA-Seq data and the corresponding clinical annotations.

The Gaussian kernel is:

$$k_{G}(x_{i},x_{j})=\text{exp}(-{\left(x_{i}-x_{j}\right)}^{⊤}(x_{i}-x_{j})/(2\sigma^{2})),$$

where *σ* is the kernel width parameter. The Fourier transform of the Gaussian kernel is:

$$p(\omega )=\sqrt{2\pi }\sigma e^{-||\omega |{|}_{2}^{2}\;\sigma^{2}/2}.$$

It is worth noting here that the Fourier transform of a Gaussian function has also Gaussian distribution. Additionally, the kernel width parameter *σ* in time space corresponds to $\sigma^{-1}$ in Fourier space.

Algorithm 1 Fast computation of kernel approximation matrix using random Fourier features

**Input:** A matrix $X\in R^{N\times d}$, a positive definite shift-invariant kernel, dimension *D* (D≪N) to calculate random Fourier features, subset size *S* (S≪N) to calculate the distance matrix.

**Output:** Low-dimensional kernel approximation matrix $Z\in R^{N\times 2D}$.

Draw *S* random rows from **X**.

Compute the Euclidean distance matrix $S\in R^{S\times S}$.

Calculate the kernel width parameter *σ* as the mean of the distance matrix **S**.

Draw *D* i.i.d. random samples $\delta_{1},\delta_{2},\ldots ,\delta_{D}\in R^{d}$ from p(·) using (1).

Draw *D* i.i.d. random samples $b_{1},b_{2},\ldots ,b_{D}\in R$ from the uniform distribution on [0, 2*π*].

Compute **Z** as:

$$\begin{array}{c} Z=\sqrt{\frac{1}{D}}[\text{cos}(\delta_{1}^{⊤}X+b_{1})\;\text{cos}(\delta_{2}^{⊤}X+b_{2})\ldots \;\text{cos}(\delta_{D}^{⊤}X+b_{D})\\ \;\text{sin}(\delta_{1}^{⊤}X+b_{1})\;\text{sin}(\delta_{2}^{⊤}X+b_{2})\ldots \;\text{sin}(\delta_{D}^{⊤}X+b_{D})]. \end{array}$$

#### 3.1.2 Fast integration with group Lasso

To be used in an MKL setting, we integrated the kernel approximations into the group Lasso formulation. After partitioned the input data according to feature sets, we computed distinct kernel matrix approximations for each feature set. Then, we concatenated the approximation matrices and used the concatenated matrix as an input to the group Lasso formulation.

The group Lasso minimizes the following objective function with respect to model parameters $\beta_{0},\beta_{1}\in R^{d_{1}},\beta_{2}\in R^{d_{2}},\ldots ,\beta_{P}\in R^{d_{P}}$:

(2) $$\sum_{i=1}^{N}\ell \left(y_{i},\beta_{0}+\sum_{p=1}^{P}x_{ip}^{⊤}\beta_{p}\right)+\lambda \sum_{p=1}^{P}\sqrt{d_{p}}||\beta_{p}|{|}_{2},$$

where ℓ(·,·) denotes the loss function according to problem type (e.g. square loss for regression problems, logistic loss for binary classification problems), *y_i_* is the output for *i*th data point (i.e. response value for regression problems, class label for binary classification problems), *λ* is a positive regularization coefficient, *d_p_* refers to the size of feature set *p* and $||\cdot |{|}_{2}$ is the Euclidean norm. This optimization problem is convex with respect to the weights $\beta_{p}$ associated with feature sets. The fact that the Euclidean norm of a vector is zero only if all vector entries are zero, the group Lasso formulation leads to sparse solutions at group level, which facilitates the feature set selection process.

**Fig. 1.**
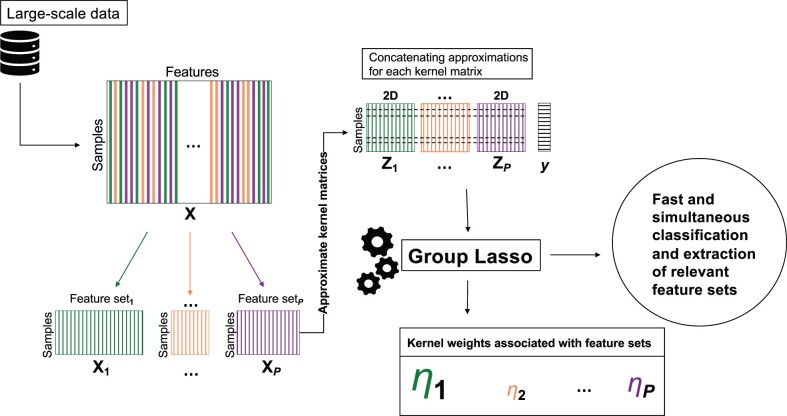
Our proposed MAKL algorithm that integrates kernel approximation and group Lasso formulations into a conjoint model. It takes the input matrix $X\in R^{N\times d}$, a binary outcome vector ***y*** of length *N*, and a pathway/gene set collection consisting of *P* pathways/gene sets, as its inputs. It then computes distinct kernel approximation matrices for each pathway/gene set, denoted as $Z_{1},Z_{2},\ldots ,Z_{P}$; and finally combine all kernel approximation matrices to get $Z\in R^{N\times 2DP}$, to be used in the group Lasso formulation. During learning process, it determines the weights associated with each pathway/gene set, denoted as $\eta_{1},\ldots ,\eta_{P}$. It is scalable since it integrates a low-dimensional kernel matrix approximation instead of usual kernel matrix computation; it is interpretable since it performs feature selection and sorts pathway/gene sets according to their relevance by comparing their $\ell_{2}$-norms

Overall, our algorithm for MKL using any kernel approximation method is described in Algorithm 2 with details. Figure 1 displays an overview of our proposed algorithm that combines Algorithms 1 and 2.

Algorithm 2 Fast integration of approximated kernels into group Lasso

**Input:** Training matrix $X\in R^{N\times d}$, *P* feature sets $F_{1},F_{2},\ldots ,F_{P}$, binary output vector ***y*** of length *N*.

**Output:** Classification area under the receiver operating characteristics curve (AUROC) value, $\ell_{2}$-norm of the weights assigned to $F_{p}$ denoted as *η_p_* for all p∈P.

Split the training matrix into *P* partitions.

Compute low-dimensional approximation matrices $Z_{1},Z_{2},\ldots ,Z_{P}$.

Concatenate distinct approximation matrices $Z_{1},Z_{2},\ldots ,Z_{P}$ to obtain one single matrix $Z\in R^{N\times 2DP}$.

Solve the group Lasso formulation using **Z** as the input matrix.

Calculate $\ell_{2}$-norm using the weights of each $F_{p}$ and denote the corresponding norm calculated for $F_{p}$ as *η_p_* for all p∈P.

If *η_p_* is non-zero, count $F_{p}$ selected for the classification task.

## 4 Results

To test our algorithm, we performed computational experiments for three binary classification tasks. To better compare the outcomes of the experiments, we benchmarked all algorithms over 100 independent replications. As the predictive performance metric for these binary classification tasks, we used AUROC, whose larger values correspond to a better classification performance.

Owing to the serious scalability issues presented at earlier sections, we did not choose standard MKL algorithms for baseline comparison. We chose extreme gradient boosting (XGBoost; Chen and Guestrin, 2016) as the baseline algorithm to compare against our Multiple Approximate Kernel Learning (MAKL) algorithm. Extreme gradient boosting is an ensemble of weak prediction models and, by its nature, its predictive performance is generally better compared with the random forest algorithm. It is also known as being scalable.

For all experiments, we split the corresponding dataset into two parts, in a way that 80% of the samples were included into the training set and the remaining 20% of the samples were included into the test set. We normalized each feature in the training set to have zero mean and unit SD. For the test set, we normalized each feature with the mean and SD calculated on the original training set. We performed 4-fold inner cross-validation on the training set for hyper-parameter tuning. We used two aforementioned pathway/gene set collections.

For XGBoost algorithm that we used as the baseline algorithm, the hyper-parameter, maximum depth of a tree, was chosen using 4-fold inner cross-validation on the training set with a maximum number of iterations of 1000 and learning rate of 0.2. Since, in this article, we cover binary classification tasks, we used ‘binary: logistic’ option as the objective function argument to the algorithm. For XGBoost experiments, we used XGBoost R package (Chen and He, 2019).

The hyper-parameter related to the regularization, *λ*, for MAKL was chosen using four-fold cross-validation on the training set. The *λ* parameter set used for cross-validation was {0.9,0.8,0.7,0.6} to get fast and sparse solutions, since parameter values closer to 1 leads to sparse solutions.

The dimensionality parameter *D* (i.e. the number of random features used to approximate distinct kernel matrices) was given as an input to the MAKL algorithm. We ran experiments using different *D* values, {100,150,200,250} to perform sensitivity analysis on MAKL. For MAKL experiments, we used grplasso R package with default parameters (Meier, 2020).

### 4.1 Early- and late-stage cancer classification

For this binary classification task, we performed sensitivity analysis for the AUROC values using different *D* values for MAKL with Hallmark gene sets (i.e. different numbers of randomly chosen Fourier samples). We compared MAKL algorithm against the baseline algorithms, XGBoost (Subset) and XGBoost (Full). XGBoost (Subset) denotes the algorithm is trained using the genes from the Hallmark gene set collection, whereas XGBoost (Full) denotes the algorithm is trained using all available 19 814 genes.

**Fig. 2.**
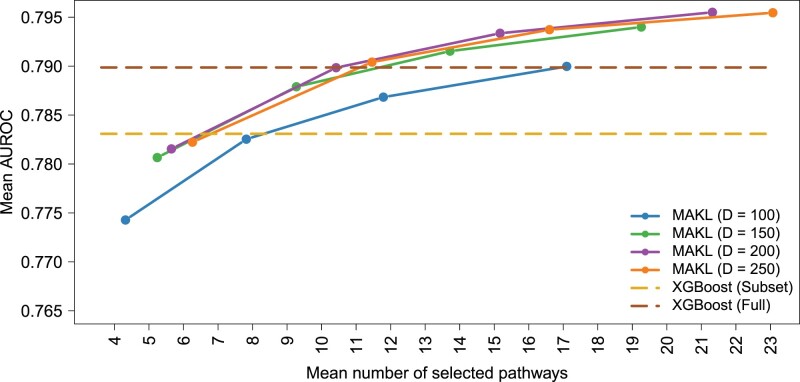
Sensitivity analysis on the early- and late-stage cancer classification performance of MAKL using four different *D* values (i.e. different numbers of randomly chosen Fourier samples), and their comparison to the baseline algorithms, XGBoost (Subset) and XGBoost (Full). The figure depicts the AUROC values resulted from 100 replications. As feature sets, the Hallmark gene set collection is used. XGBoost (Subset) denotes that the algorithm is trained using the genes from the Hallmark gene set collection and XGBoost (Full) denotes that the algorithm is trained using all available 19 814 genes. Note that the results reported for XGBoost (Subset) and XGBoost (Full) are not affected from the values in the *x*-axis, and they are reported as dashed lines for easy comparison

Figure 2 shows how the classification performance changes with respect to the number of feature sets (i.e. pathway/gene sets) used. It also shows that MAKL with Hallmark gene set collection outperforms XGBoost (Subset) using only a small fraction of the available features. Considering the mean number of pathways used for classification, MAKL outperforms the baseline algorithm XGBoost (Subset) using only 18.54% (i.e. 9.27 out of 50) of the total feature sets (i.e. gene sets) with *D *=* *150. It also outperformed XGBoost (Full) while using only 12.47% of the all available 19 814 genes on the average. Additionally, Supplementary Figure S1 displays the early- and late-stage cancer classification sensitivity analysis for the AUROC values using different *D* values for MAKL with PID pathway collection and compares MAKL against the baseline algorithms, namely, XGBoost (Subset) and XGBoost (Full).

### 4.2 Two-year survival classification

For 2-year survival classification task, MAKL with PID pathway collection was able to outperform the baseline algorithms XGBoost (Subset) using only 5.96% (i.e. 11.69 out of 196) of the total feature sets (i.e. pathways) with *D *=* *150. It also outperformed XGBoost (Full) while using only 4.15% of all available 19 814 genes on the average.

**Fig. 3.**
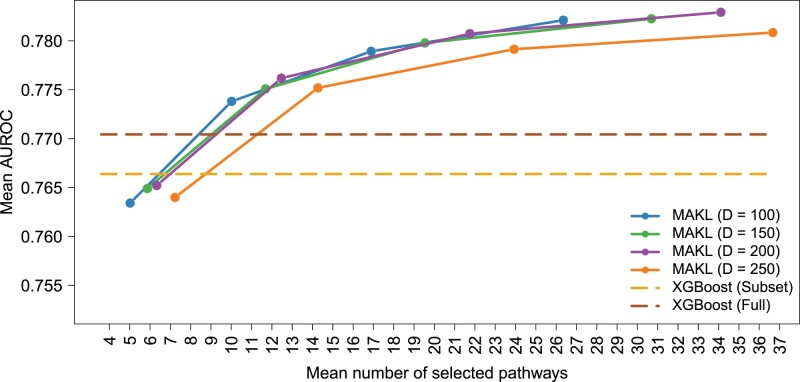
Sensitivity analysis on the 2-year survival classification performance of MAKL using four different *D* values (i.e. different numbers of randomly chosen Fourier samples), and their comparison to the baseline algorithms, XGBoost (Subset) and XGBoost (Full). The figure depicts the AUROC values resulted from 100 replications. As feature sets, the PID pathway collection is used. XGBoost (Subset) denotes that the algorithm is trained using the genes from the PID pathway collection, and XGBoost (Full) denotes that the algorithm is trained using all available 19 814 genes. Note that the results reported for XGBoost (Subset) and XGBoost (Full) are not affected from the values in the *x*-axis, and they are reported as dashed lines for easy comparison

Figure 3 shows sensitivity analysis for the AUROC values using four different *D* values for MAKL with PID pathway collection (i.e. different numbers of randomly chosen Fourier samples), and compares them against the baseline algorithms, XGBoost (Subset) and XGBoost (Full). XGBoost (Subset) denotes the algorithm is trained using the genes from the PID pathway collection, and XGBoost (Full) denotes the algorithm is trained using all available 19 814 genes. Additionally, Supplementary Figure S2 shows the sensitivity analysis for the AUROC values using four different *D* values of MAKL with Hallmark gene sets and compares the results with the baseline algorithms, XGBoost (Subset) and XGBoost (Full). XGBoost (Subset) denotes the algorithm is trained using the genes from the Hallmark gene set collection, and XGBoost (Full) denotes the algorithm is trained using all available 19 814 genes.

### 4.3 Single-cell melanoma immunotherapy dataset

As mentioned earlier, our algorithm is capable of extracting meaningful information while learning a predictive model. The weights associated with each pathway/gene set resulting from MAKL show the relevance of each pathway in the given classification task. Owing to the computational pre-processing and labelling techniques used in single-cell dataset that we used (i.e. the labelling is not done manually and by observation like in patient labelling), we concentrated on the pathways/gene sets that play crucial role in cell malignancy classification task, rather than the classification performance. We demonstrated that the pathways/gene sets that our algorithm MAKL found important for this classification task are relevant in terms of finding new immunotherapy techniques, new ways to discover tumour heterogeneity since the pathways/gene sets extracted from our algorithm are in line with the most recent melanoma treatment trials, which are supported with the literature findings as detailed below.

Supplementary Table S1 displays the selection frequencies of 50 gene sets in the Hallmark gene set collection for over 100 replications, using four different regularization parameters (i.e. *λ* multipliers). If norm of weights associated with a feature set, resulting from MAKL is positive, we considered the corresponding feature set (i.e. pathway/gene set) as selected. Supplementary Table S2 displays the selection frequencies of 196 pathways in the PID pathway collection for 100 replications, using four different regularization parameters (i.e. *λ* multipliers). According to the average selection frequencies over 100 replications, the most selected pathway by MAKL with PID pathway collection was CXCR4 pathway. This result is relevant since targeting of CXCR4 pathway is under consideration in melanoma treatment trials. Supplementary Table S2 also shows that, even using four pathways, MAKL is capable of finding this relevant pathway for cell malignancy classification. In other words, our algorithm can find the relevant feature sets towards a classification task using a small fraction of all the feature sets (e.g. 2.26 out of 196).

It is stated in literature that targeting melanoma by CXCR4 inhibition may be a good way to destroy the tumour cells and that CXCR4 regulates tumour immunity (Yang *et al.*, 2018). Additionally, the chemokine receptor CXCR4 is stated to be associated with cancer growth, invasion and metastasis and identified as an independent predictor of poor prognosis in primary melanoma (Scala *et al.*, 2006). The three most frequently chosen pathways resulted from MAKL with PID pathway collection over 100 independent replications are CXCR4, SYNDECAN_4 and CASPASE, respectively. Likewise, the three most frequently chosen pathways resulted from MAKL with Hallmark gene set collection over 100 independent replications are ALLOGRAFT_REJECTION, INTERFERON_ALPHA_RESPONSE and KRAS_SIGNALING_UP, respectively. Further related details are given in Supplementary Tables S1 and S2.

## 5 Conclusions

In this article, we introduced a scalable MAKL, which is designed specifically for large-scale genomic datasets. Our approach can combine any low-dimensional kernel approximation with a group Lasso formulation. We used a modified version of the random feature mapping as kernel approximation algorithm in our experiments (see Algorithms 1 and 2 for details). MAKL is fast and appropriate for large-scale genomic data such as single-cell sequencing data having large sample-size with many features. Our method can integrate prior information in the form of gene sets/pathways into the learning process to increase the interpretability without sacrificing the predictive accuracy.

To benchmark our algorithm, we used three datasets constructed from two data sources. As prior information sources, we used Hallmark and PID pathway/gene set collections particularly curated for cancer research. We processed genomic data from TCGA project to form the early- and late-stage cancer dataset together with 2-year cancer survival dataset. We also used single-cell RNA-Seq data provided by the Broad Institute Single Cell Portal to understand the molecular underpinnings of cell malignancy. Our algorithm MAKL was capable of outperforming the baseline algorithms based on XGBoost using only a small fraction of the pathway/gene sets available (see Figs 2 and 3, Supplementary Figs S1 and S2); thus, it provided sparse solutions. It also provided selection frequencies associated with the pathway/gene sets used as prior information sources for the corresponding classification task (Supplementary Tables S1 and S2), which can be used to understand the biological mechanisms towards the applied classification problem. Regarding scalability, MAKL was trained (while simultaneously extracting the significant information from it, unlike the baseline algorithms) <30 s on the average for the three problems we discussed while the baseline algorithms XGBoost (Subset) and XGBoost (Full) perform the same classification task between 1 and 4 min on the average, respectively. This scalability property, unlike the standard MKL algorithms, makes it possible for MAKL to be used with large-scale data, such as single-cell genomic datasets. As a result, this characteristic could lead to discovery of new biomarkers for tumour plasticity and of new techniques for immunotherapy and targeted cancer therapies.

An interesting direction for future studies may be building a multi-class extension of MAKL to be used with multi-class problems. Also, integration of low-dimensional kernel approximation into other machine learning models is exciting considering the increasing need for fast data processing and interpretability. Our approach is promising in domains including but not limited to computational biology and computer vision thanks to its scalability, flexibility and also its ability to deal with highly correlated features.

## Funding

This work was supported by the Scientific and Technological Research Council of Turkey (TÜBİTAK) [EEEAG 117E181]. M.G. was supported by the Turkish Academy of Sciences (TÜBA-GEBİP; The Young Scientist Award Program) and the Science Academy of Turkey (BAGEP; The Young Scientist Award Program).

*Conflict of Interest*: none declared.

## Supplementary Material

btac241_Supplementary_Data
