# Supplementary material for: Fast and interpretable genomic data analysis using multiple approximate kernel learning
Source: Bioinformatics. 2022 Jun 27;38(Suppl 1):i77–83. doi: 10.1093/bioinformatics/btac241 (PMC9235505; doi:10.1093/bioinformatics/btac241)
Supplement: btac241_Supplementary_Data [file btac241_supplementary_data.pdf]

Fast and interpretable genomic data analysis using multiple approximate kernel learning — Supplementary material —

Ayyüce Begüm Bektaş[1], Çiğdem Ak[2], and Mehmet Gönen[3,4]

[1]Graduate School of Sciences and Engineering, Koç University, İstanbul 34450, Turkey
[2]Knight Cancer Institute, Oregon Health & Science University, Portland, OR 97239, USA
[3]Department of Industrial Engineering, College of Engineering, Koç University, İstanbul 34450, Turkey
[4]School of Medicine, Koç University, İstanbul 34450, Turkey

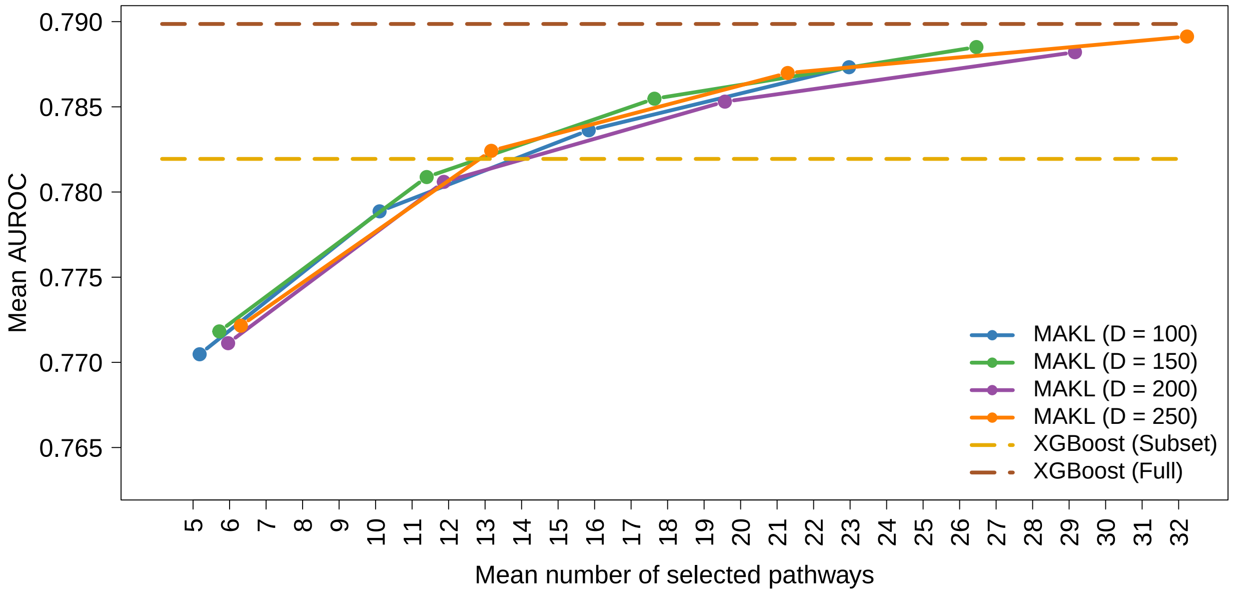

Supplementary Fig. 1. Sensitivity analysis on the early- and late-stage cancer classification performance of MAKL using four different D values (i.e. different numbers of randomly chosen Fourier samples), and their comparison to the baseline algorithms, XGBoost (Subset) and XGBoost (Full). The figure depicts the AUROC values resulted from 100 replications. As feature sets, the Pathway Interaction Database pathway collection (PID) is used. XGBoost (Subset) denotes that the algorithm is trained using the genes from the PID gene set collection, and XGBoost (Full) denotes that the algorithm is trained using all available 19814 genes. Note that the results reported for XGBoost (Subset) and XGBoost (Full) are not affected from the values in the x-axis, and they are reported as dashed lines for easy comparison.

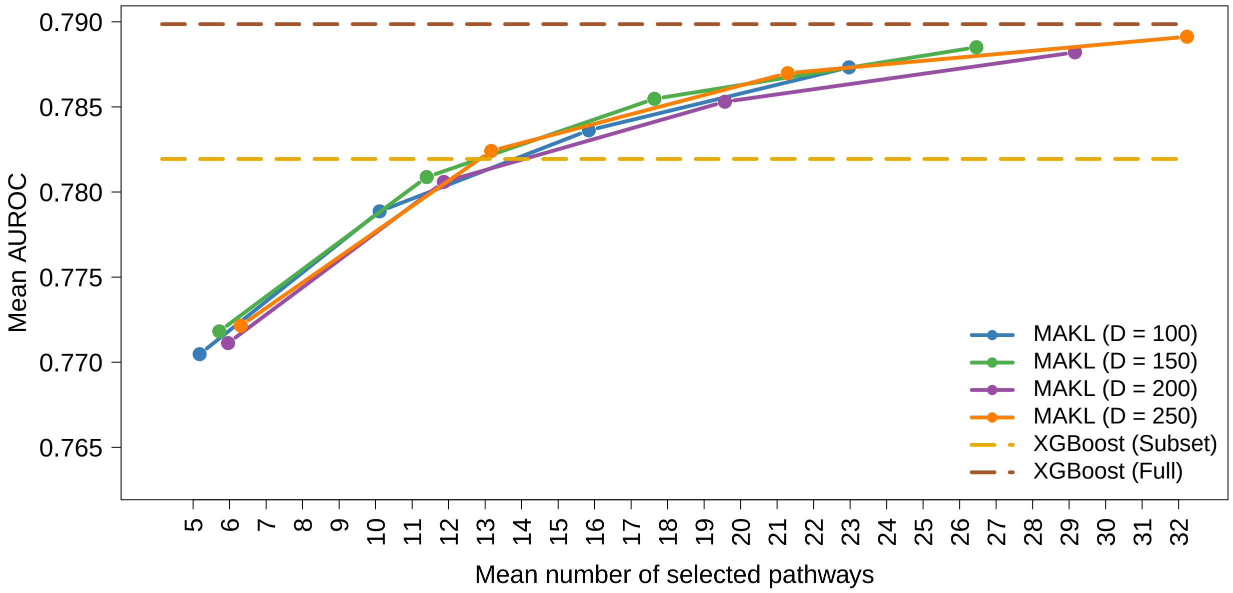

Supplementary Fig. 2. Sensitivity analysis on the two-year survival classification performance of MAKL using four different D values (i.e. different numbers of randomly chosen Fourier samples), and their comparison to the baseline algorithms, XGBoost (Subset) and XGBoost (Full). The figure depicts the AUROC values resulted from 100 replications. As feature sets, Hallmark gene set collection is used. XGBoost (Subset) denotes that the algorithm is trained using the genes from the Hallmark gene set collection, and XGBoost (Full) denotes that the algorithm is trained using all available 19814 genes. Note that the results reported for XGBoost (Subset) and XGBoost (Full) are not affected from the values in the x-axis, and they are reported as dashed lines for easy comparison.

Supplementary Table 1. Selection frequencies of 50 gene sets in the Hallmark collection by MAKL algorithm over 100 replications using single-cell melanoma immunotherapy dataset, for detailed pathway and gene analysis regarding the cell malignancy classification task. Selection frequencies using 4 different regularization parameters (i.e. λ multiplier; please see the main manuscript for details) with $D = 100$ are reported. At the last column, the mean selection frequency for each gene set is reported and the gene sets are sorted from the most selected one to the least selected one.

	λ				Mean selection
**Gene set name**	**0.9**	**0.8**	**0.7**	**0.6**	**frequency**
ALLOGRAFT_REJECTION	95	98	100	100	98.25
INTERFERON_ALPHA_RESPONSE	67	86	95	96	86.00
KRAS_SIGNALING_UP	52	78	95	99	81.00
EPITHELIAL_MESENCHYMAL_TRANSITION	24	52	76	91	60.75
APOPTOSIS	25	52	75	87	59.75
ANDROGEN_RESPONSE	17	46	76	94	58.25
INTERFERON_GAMMA_RESPONSE	13	30	36	52	32.75
APICAL_JUNCTION	5	19	36	51	27.75
COAGULATION	1	2	8	22	8.25
IL2_STAT5_SIGNALING	0	2	8	15	6.25
MYOGENESIS	2	2	5	8	4.25
HYPOXIA	0	0	3	9	3.00
COMPLEMENT	0	1	1	4	1.50
INFLAMMATORY_RESPONSE	0	0	0	0	0
ESTROGEN_RESPONSE_LATE	0	0	0	0	0
ESTROGEN_RESPONSE_EARLY	0	0	0	0	0
MTORC1_SIGNALING	0	0	0	0	0
GLYCOLYSIS	0	0	0	0	0
UV_RESPONSE_DN	0	0	0	0	0
APICAL_SURFACE	0	0	0	0	0
TNFA_SIGNALING_VIA_NFKB	0	0	0	0	0
REACTIVE_OXIGEN_SPECIES_PATHWAY	0	0	0	0	0
HEME_METABOLISM	0	0	0	0	0
UV_RESPONSE_UP	0	0	0	0	0
P53_PATHWAY	0	0	0	0	0
ADIPOGENESIS	0	0	0	0	0
XENOBIOTIC_METABOLISM	0	0	0	0	0
PI3K_AKT_MTOR_SIGNALING	0	0	0	0	0
IL6_JAK_STAT3_SIGNALING	0	0	0	0	0
OXIDATIVE_PHOSPHORYLATION	0	0	0	0	0
PANCREAS_BETA_CELLS	0	0	0	0	0
CHOLESTEROL_HOMEOSTASIS	0	0	0	0	0
MITOTIC_SPINDLE	0	0	0	0	0
WNT_BETA_CATENIN_SIGNALING	0	0	0	0	0
TGF_BETA_SIGNALING	0	0	0	0	0
DNA_REPAIR	0	0	0	0	0
G2M_CHECKPOINT	0	0	0	0	0
NOTCH_SIGNALING	0	0	0	0	0
PROTEIN_SECRETION	0	0	0	0	0
HEDGEHOG_SIGNALING	0	0	0	0	0

UNFOLDED_PROTEIN_RESPONSE	0	0	0	0	0
E2F_TARGETS	0	0	0	0	0
MYC_TARGETS_V1	0	0	0	0	0
MYC_TARGETS_V2	0	0	0	0	0
FATTY_ACID_METABOLISM	0	0	0	0	0
ANGIOGENESIS	0	0	0	0	0
BILE_ACID_METABOLISM	0	0	0	0	0
PEROXISOME	0	0	0	0	0
SPERMATOGENESIS	0	0	0	0	0
KRAS_SIGNALING_DN	0	0	0	0	0

Supplementary Table 2. Selection frequencies of 196 pathways in the PID collection by MAKL algorithm over 100 replications using single-cell melanoma immunotherapy dataset, for detailed pathway and gene analysis regarding the cell malignancy classification task. Selection frequencies using 4 different regularization parameters (i.e. λ multiplier; please see the main manuscript for details) with $D = 100$ are reported. At the last column, the mean selection frequency for each gene set is reported and the gene sets are sorted from the most selected one to the least selected one.

**Pathway name**	λ **0.9**	**0.8**	**0.7**	**0.6**	**Mean selection frequency**
CXCR4_PATHWAY	100	100	100	100	100.00
SYNDECAN_4_PATHWAY	70	96	100	100	91.50
CASPASE_PATHWAY	55	100	100	100	88.75
IL12_2PATHWAY	1	23	77	98	49.75
BETA_CATENIN_NUC_PATHWAY	0	1	25	74	25.00
PDGFRB_PATHWAY	0	8	30	51	22.25
IL12_STAT4_PATHWAY	0	5	18	48	17.75
AP1_PATHWAY	0	1	4	44	12.25
TCR_PATHWAY	0	2	6	12	5.00
IL4_2PATHWAY	0	1	1	13	3.75
ECADHERIN_NASCENT_AJ_PATHWAY	0	0	1	11	3.00
ARF6_TRAFFICKING_PATHWAY	0	0	3	8	2.75
CD8_TCR_PATHWAY	0	0	0	5	1.25
P53_DOWNSTREAM_PATHWAY	0	0	0	1	0.25
INTEGRIN1_PATHWAY	0	0	0	0	0
AVB3_INTEGRIN_PATHWAY	0	0	0	0	0
SYNDECAN_1_PATHWAY	0	0	0	0	0
CMYB_PATHWAY	0	0	0	0	0
HIF1_TFPATHWAY	0	0	0	0	0
MYC_ACTIV_PATHWAY	0	0	0	0	0
NCADHERIN_PATHWAY	0	0	0	0	0
INTEGRIN2_PATHWAY	0	0	0	0	0
ILK_PATHWAY	0	0	0	0	0
MYC_REPRESS_PATHWAY	0	0	0	0	0
RHOA_REG_PATHWAY	0	0	0	0	0
INTEGRIN3_PATHWAY	0	0	0	0	0
RB_1PATHWAY	0	0	0	0	0
INTEGRIN_A4B1_PATHWAY	0	0	0	0	0
FGF_PATHWAY	0	0	0	0	0
AJDISS_2PATHWAY	0	0	0	0	0
VEGFR1_2_PATHWAY	0	0	0	0	0
S1P_S1P3_PATHWAY	0	0	0	0	0
PI3KCI_PATHWAY	0	0	0	0	0
ENDOTHELIN_PATHWAY	0	0	0	0	0
FOXM1_PATHWAY	0	0	0	0	0
FCER1_PATHWAY	0	0	0	0	0
SYNDECAN_3_PATHWAY	0	0	0	0	0
ERBB1_DOWNSTREAM_PATHWAY	0	0	0	0	0
EPHRINB_REV_PATHWAY	0	0	0	0	0
ALPHA_SYNUCLEIN_PATHWAY	0	0	0	0	0

PTP1B_PATHWAY	0	0	0	0	0
P38_MK2_PATHWAY	0	0	0	0	0
A6B1_A6B4_INTEGRIN_PATHWAY	0	0	0	0	0
IL2_1PATHWAY	0	0	0	0	0
DELTA_NP63_PATHWAY	0	0	0	0	0
AURORA_B_PATHWAY	0	0	0	0	0
CD8_TCR_DOWNSTREAM_PATHWAY	0	0	0	0	0
P73PATHWAY	0	0	0	0	0
SHP2_PATHWAY	0	0	0	0	0
P38_ALPHA_BETA_DOWNSTREAM_PATHWAY	0	0	0	0	0
ECADHERIN_STABILIZATION_PATHWAY	0	0	0	0	0
FANCONI_PATHWAY	0	0	0	0	0
SMAD2_3NUCLEAR_PATHWAY	0	0	0	0	0
BCR_5PATHWAY	0	0	0	0	0
PRL_SIGNALING_EVENTS_PATHWAY	0	0	0	0	0
RHOA_PATHWAY	0	0	0	0	0
ERBB4_PATHWAY	0	0	0	0	0
LYSOPHOSPHOLIPATHWAY	0	0	0	0	0
INSULIN_PATHWAY	0	0	0	0	0
NOTCH_PATHWAY	0	0	0	0	0
P38_MKK3_6PATHWAY	0	0	0	0	0
GMCSF_PATHWAY	0	0	0	0	0
WNT_NONCANONICAL_PATHWAY	0	0	0	0	0
NFKAPPAB_ATYPICAL_PATHWAY	0	0	0	0	0
HDAC_CLASSII_PATHWAY	0	0	0	0	0
BETA_CATENIN_DEG_PATHWAY	0	0	0	0	0
HDAC_CLASSIII_PATHWAY	0	0	0	0	0
GLYPICAN_1PATHWAY	0	0	0	0	0
IL27_PATHWAY	0	0	0	0	0
NFKAPPAB_CANONICAL_PATHWAY	0	0	0	0	0
E2F_PATHWAY	0	0	0	0	0
ER_NONGENOMIC_PATHWAY	0	0	0	0	0
DNA_PK_PATHWAY	0	0	0	0	0
HIF2PATHWAY	0	0	0	0	0
CD40_PATHWAY	0	0	0	0	0
ATR_PATHWAY	0	0	0	0	0
INTEGRIN_CS_PATHWAY	0	0	0	0	0
MET_PATHWAY	0	0	0	0	0
LPA4_PATHWAY	0	0	0	0	0
AR_PATHWAY	0	0	0	0	0
NFAT_TFPATHWAY	0	0	0	0	0
EPHB_FWD_PATHWAY	0	0	0	0	0
AVB3_OPN_PATHWAY	0	0	0	0	0
S1P_S1P4_PATHWAY	0	0	0	0	0
FRA_PATHWAY	0	0	0	0	0
REELIN_PATHWAY	0	0	0	0	0
PS1_PATHWAY	0	0	0	0	0
NECTIN_PATHWAY	0	0	0	0	0
P38_ALPHA_BETA_PATHWAY	0	0	0	0	0
WNT_SIGNALING_PATHWAY	0	0	0	0	0

TRAIL_PATHWAY	0	0	0	0	0
CDC42_PATHWAY	0	0	0	0	0
RET_PATHWAY	0	0	0	0	0
CDC42_REG_PATHWAY	0	0	0	0	0
ATM_PATHWAY	0	0	0	0	0
ARF6_PATHWAY	0	0	0	0	0
LKB1_PATHWAY	0	0	0	0	0
WNT_CANONICAL_PATHWAY	0	0	0	0	0
TCPTP_PATHWAY	0	0	0	0	0
ANGIOPOIETIN_RECEPTOR_PATHWAY	0	0	0	0	0
FAS_PATHWAY	0	0	0	0	0
CIRCADIAN_PATHWAY	0	0	0	0	0
TXA2PATHWAY	0	0	0	0	0
HDAC_CLASSI_PATHWAY	0	0	0	0	0
S1P_S1P1_PATHWAY	0	0	0	0	0
TELOMERASE_PATHWAY	0	0	0	0	0
HNF3B_PATHWAY	0	0	0	0	0
NETRIN_PATHWAY	0	0	0	0	0
IL1_PATHWAY	0	0	0	0	0
NFAT_3PATHWAY	0	0	0	0	0
REG_GR_PATHWAY	0	0	0	0	0
CONE_PATHWAY	0	0	0	0	0
INTEGRIN_A9B1_PATHWAY	0	0	0	0	0
ERB_GENOMIC_PATHWAY	0	0	0	0	0
ARF6_DOWNSTREAM_PATHWAY	0	0	0	0	0
MTOR_4PATHWAY	0	0	0	0	0
IGF1_PATHWAY	0	0	0	0	0
ERBB1_RECEPTOR_PROXIMAL_PATHWAY	0	0	0	0	0
TNF_PATHWAY	0	0	0	0	0
PLK1_PATHWAY	0	0	0	0	0
TCR_RAS_PATHWAY	0	0	0	0	0
IL5_PATHWAY	0	0	0	0	0
FOXO_PATHWAY	0	0	0	0	0
VEGF_VEGFR_PATHWAY	0	0	0	0	0
THROMBIN_PAR4_PATHWAY	0	0	0	0	0
MYC_PATHWAY	0	0	0	0	0
RANBP2_PATHWAY	0	0	0	0	0
IL2_PI3K_PATHWAY	0	0	0	0	0
CERAMIDE_PATHWAY	0	0	0	0	0
AR_TF_PATHWAY	0	0	0	0	0
P75_NTR_PATHWAY	0	0	0	0	0
S1P_META_PATHWAY	0	0	0	0	0
INTEGRIN4_PATHWAY	0	0	0	0	0
AMB2_NEUTROPHILS_PATHWAY	0	0	0	0	0
IFNG_PATHWAY	0	0	0	0	0
RXR_VDR_PATHWAY	0	0	0	0	0
LIS1_PATHWAY	0	0	0	0	0
ATF2_PATHWAY	0	0	0	0	0
UPA_UPAR_PATHWAY	0	0	0	0	0
ERBB2_ERBB3_PATHWAY	0	0	0	0	0

EPHA_FWDPATHWAY	0	0	0	0	0
HIF1A_PATHWAY	0	0	0	0	0
BMP_PATHWAY	0	0	0	0	0
IL3_PATHWAY	0	0	0	0	0
IL6_7_PATHWAY	0	0	0	0	0
ECADHERIN_KERATINOCYTE_PATHWAY	0	0	0	0	0
ALK1_PATHWAY	0	0	0	0	0
TRKR_PATHWAY	0	0	0	0	0
TCR_JNK_PATHWAY	0	0	0	0	0
NEPHRIN_NEPH1_PATHWAY	0	0	0	0	0
IL23_PATHWAY	0	0	0	0	0
HIV_NEF_PATHWAY	0	0	0	0	0
ERA_GENOMIC_PATHWAY	0	0	0	0	0
ERBB_NETWORK_PATHWAY	0	0	0	0	0
ALK2_PATHWAY	0	0	0	0	0
RHODOPSIN_PATHWAY	0	0	0	0	0
PDGFRA_PATHWAY	0	0	0	0	0
RETINOIC_ACID_PATHWAY	0	0	0	0	0
P38_GAMMA_DELTA_PATHWAY	0	0	0	0	0
IL8_CXCR2_PATHWAY	0	0	0	0	0
HEDGEHOG_2PATHWAY	0	0	0	0	0
INTEGRIN5_PATHWAY	0	0	0	0	0
AR_NONGENOMIC_PATHWAY	0	0	0	0	0
ERBB1_INTERNALIZATION_PATHWAY	0	0	0	0	0
HEDGEHOG_GLI_PATHWAY	0	0	0	0	0
CXCR3_PATHWAY	0	0	0	0	0
VEGFR1_PATHWAY	0	0	0	0	0
SMAD2_3PATHWAY	0	0	0	0	0
KIT_PATHWAY	0	0	0	0	0
EPO_PATHWAY	0	0	0	0	0
IL2_STAT5_PATHWAY	0	0	0	0	0
TCR_CALCIUM_PATHWAY	0	0	0	0	0
THROMBIN_PAR1_PATHWAY	0	0	0	0	0
SYNDECAN_2_PATHWAY	0	0	0	0	0
RAC1_REG_PATHWAY	0	0	0	0	0
AURORA_A_PATHWAY	0	0	0	0	0
ARF_3PATHWAY	0	0	0	0	0
INSULIN_GLUCOSE_PATHWAY	0	0	0	0	0
PI3KCI_AKT_PATHWAY	0	0	0	0	0
IL8_CXCR1_PATHWAY	0	0	0	0	0
TAP63_PATHWAY	0	0	0	0	0
BARD1_PATHWAY	0	0	0	0	0
P53_REGULATION_PATHWAY	0	0	0	0	0
TOLL_ENDOGENOUS_PATHWAY	0	0	0	0	0
ANTHRAX_PATHWAY	0	0	0	0	0
S1P_S1P2_PATHWAY	0	0	0	0	0
RAS_PATHWAY	0	0	0	0	0
MAPK_TRK_PATHWAY	0	0	0	0	0
PI3K_PLC_TRK_PATHWAY	0	0	0	0	0
EPHA2_FWD_PATHWAY	0	0	0	0	0

LYMPH_ANGIOGENESIS_PATHWAY	0	0	0	0	0
RAC1_PATHWAY	0	0	0	0	0
FAK_PATHWAY	0	0	0	0	0
HNF3A_PATHWAY	0	0	0	0	0
TGFBR_PATHWAY	0	0	0	0	0
HES_HEY_PATHWAY	0	0	0	0	0
